# Angioplasty and Stenting of Intracranial Arterial Stenosis in Perforator-Bearing Segments: A Comparison Between the Anterior and the Posterior Circulation

**DOI:** 10.3389/fneur.2018.00533

**Published:** 2018-07-09

**Authors:** Hannes Nordmeyer, René Chapot, Ayhan Aycil, Christian P. Stracke, Marta Wallocha, M. Jeffrie Hadisurya, Markus Heddier, Patrick Haage, Ralph Weber

**Affiliations:** ^1^Department of Radiology and Neuroradiology, Alfried Krupp Krankenhaus Essen, Essen, Germany; ^2^Faculty of Health, School of Medicine, Witten/Herdecke University, Witten, Germany; ^3^Gemeinschaftspraxis Aycil/Kilicli, Mülheim an der Ruhr, Germany; ^4^Department of Diagnostic and Interventional Neuroradiology, University Medical Center Hamburg-Eppendorf, Hamburg, Germany; ^5^Department of Neurology, Alfried Krupp Krankenhaus, Essen, Germany; ^6^Department of Diagnostic and Interventional Radiology, HELIOS University Hospital Wuppertal, Witten/Herdecke University, Wuppertal, Germany; ^7^School of Medicine, Faculty of Health, Ruhr University, Bochum, Germany

**Keywords:** intracranial stenosis, atherosclerosis, intracranial embolism and thrombosis, perforators, ischemic stroke, stenting, angioplasty, PTA

## Abstract

**Background and Purpose:** Subgroup analysis of the SAMMPRIS trial showed a higher rate of periprocedural perforator strokes with the Wingspan stent in the basilar artery in patients with symptomatic intracranial atherosclerotic stenosis (ICAS). It remains unclear whether angioplasty (PTA) alone or in combination with other stent types (PTAS) will yield similar results in perforator-bearing segments of the anterior and posterior circulation.

**Methods:** We retrospectively analyzed the periprocedural complication rate, long term outcome and stroke etiology in 59 consecutive patients with ICAS of the middle cerebral artery (79 treatments) and 67 patients with ICAS of the intracranial vertebral and basilar artery (76 treatments) treated with PTA or PTAS from 2007 to 2015 in a high-volume neuro-interventional center.

**Results:** Periprocedural symptomatic ischemic strokes occurred significantly more often in patients with posterior vs. anterior ICAS treatment (14.5 vs. 5.1%, *p* = 0.048). During a mean follow-up period of 19 (±23.7) months, 5 recurrent ischemic and 2 hemorrhagic strokes (10.4%) occurred in the territory of the treated artery in posterior circulation compared to 2 ischemic strokes in the anterior circulation (3.4%, *p* = 0.549). Overall, significantly more patients treated for a posterior ICAS suffered a periprocedural or follow-up stroke [25% vs. 11.4%, *p* = 0.024]. Periprocedural ischemic strokes were predominantly perforator strokes (73.3%), while all ischemic strokes during follow-up were caused by distal embolization (57.1%) or delayed stent occlusion (42.9%). There was no difference between PTA alone and PTAS.

**Conclusion:** The periprocedural and long-term symptomatic stroke rate was significantly higher in the treatment of perforator-bearing arteries in the posterior circulation. There was no difference between PTA alone or PTAS.

## Introduction

Symptomatic intracranial atherosclerotic stenosis (ICAS) is thought to be one of the leading causes of ischemic stroke worldwide due to a much higher prevalence in Asians compared to Caucasians (1) and bears a high risk of stroke recurrence (2). Recent randomized studies have shown a significantly higher risk of endovascular treatment of ICAS with percutaneous transluminal angioplasty and stenting (PTAS) compared to aggressive medical treatment [SAMMPRIS (3), VISSIT (4)]. A pooled analysis of the randomized SAMMPRIS and VISSIT trials (5) and an older meta-analysis of case studies found a higher risk of periprocedural stroke in treatment of ICAS in the posterior circulation (6). What is more, a detailed analysis in the SAMMPRIS trial using the Wingspan stent showed that the majority of periprocedural ischemic strokes (within 30 days after randomization) were caused by occlusion of perforator arteries (7). It was therefore hypothesized that a potential approach to reduce the risk of perforator artery occlusion by displacement of an atheroma into the artery ostium during stenting might be PTA alone (8). However, only a small randomized trial with 18 patients has compared PTA alone to PTAS in symptomatic ICAS so far (9). Furthermore, no detailed data on stroke mechanisms during long term follow-up after ICAS treatment has been published so far. In November 2016 the European Stroke Organization stated in their Karolinska Stroke Update (ESO-KSU) (10) that PTA or PTAS carried out by experienced personnel may be considered in a few special situations in patients with symptomatic ICAS (Grade C evidence) only and claimed further studies.

In order to understand interventional treatment risks of perforator-bearing brain arteries, we compared frequency and pathophysiology of periprocedural and long-term strokes and asymptomatic DWI lesions between the anterior and the posterior circulation in Caucasian patients with ICAS treated by PTA alone or PTAS with other stent types than Wingspan.

## Methods

### Patients

We retrospectively reviewed all neurointerventional angioplasty and stenting procedures from January 2007 to February 2015 and identified all interventional procedures performed in perforator-bearing segments of symptomatic ICAS (M1-segment of the MCA, V4-segment of the VA and/or BA) with documented preceding transient ischemic attack or ischemic stroke in a tertiary high-volume center. Patients with non-atherosclerotic lesions such as arterial dissection or Moyamoya angiopathy were excluded from the study.

Patient charts were then evaluated regarding age at intervention, sex, cardiovascular risk factors (hypertension, hyperlipidemia, diabetes mellitus, current smoking), atrial fibrillation, previous stroke or TIA, and time point of intervention in relation to the last TIA/stroke. Furthermore medical secondary stroke prevention (antiplatelet therapy, oral anticoagulation, statins, antihypertensives) before intervention and at discharge was recorded.

The study was approved by the local ethics committee of the University Duisburg-Essen.

### Imaging analysis

The degree of the symptomatic ICAS was analyzed on digital subtraction angiography (DSA) using the method proposed in the WASID trial (11). A brain MRI with T1-weighted, T2-weighted and diffusion-weighted sequences was performed after PTA/PTAS to investigate for new ischemic or hemorrhagic brain lesions. New diffusion-weighted brain lesions were classified as clinical overt or silent. In cases where brain MRI was not feasible (i.e., cardiac pacemaker), a post-interventional brain CT was performed.

Periprocedural or long term ischemic strokes were classified as perforator stroke due to an occlusion of a perforator-bearing artery, as embolic stroke in the distal territory of the treated artery, or as stroke caused by a local (stent) thrombotic occlusion of the treated artery. Hemorrhagic strokes were classified as subarachnoid or intracerebral hemorrhage.

### Periprocedural management and PTA/PTAS procedure

All patients received antiplatelet therapy with 100 mg aspirin and 75 mg clopidogrel daily. A clopidogrel loading dose at the day prior to the intervention was given in all patients being on a monotherapy with aspirin. Dual antiplatelet therapy was then administered for 3 months. Thereafter monotherapy with one platelet inhibitor was continued. Patients showing a non-response to clopidogrel on Multiplate® Analyzer testing (Roche Diagnostics, available from 2012) were given prasugrel or ticagrelor in addition to aspirin.

All patients were treated under general anesthesia by an experienced neuroradiologist with intra-arterial blood pressure monitoring. During the procedure 5.000 IU heparin were given intravenously right after groin puncture. The standard approach of ICAS treatment in our department is primary PTA with monorail balloons. The parent artery diameter was measured on 2D and 3D DSA images proximal and distal to the stenosis. In case of a post-stenotic dilatation, the vessel diameters proximal to the stenosis and distal to the dilatation were considered the normal diameter. In case of technical success (normalized or clearly improved hemodynamics and no vessel dissection) after PTA alone, the procedure was terminated. In cases of recoil of the stenosis or vessel dissection, the PTA was followed by stenting. Depending on the length of the stenosis and the angulation of the affected vessel, either balloon mounted coronary stents (mainly Coroflex® blue, B.Braun) or self-expanding intracranial stents (mainly Neuroform®, Stryker, and Acclino®, Acandis) were used. When self-expanding stents were used, the predominant technical approach for placement of the delivery catheter was parallel navigation along the remaining microwire that had been used for the PTA to minimize the risk of vessel perforation by exchange maneuvers.

A restenosis with recurrent symptoms or proven hemodynamic significance on control DSA or perfusion imaging (CT-Perfusion or MR-Perfusion) was treated by PTAS in case of primary treatment without stenting and by PTA with drug eluting balloons in cases of primary PTAS.

### Periprocedural complications and long-term outcome

Endovascular procedures were assessed regarding technical angiographic complication such as distal wire perforations, vessel rupture, dissection, thromboembolism, and parent or side branch artery occlusion. Neurological function was examined within 24 h after the procedure and at hospital discharge. All patients were scheduled for follow-up conventional angiography and neurological examination 6–12 months after the procedure. Patients not eligible for conventional angiography were controlled with MR or CT angiography. When no clinical long term follow-up examination had been performed, a questionnaire was sent to the patient or a telephone interview was performed to assess functional outcome (modified Rankin scale, mRS) and recurrent strokes.

### Statistical analysis

Baseline characteristics, periprocedural complications and long-term outcome parameters were compared using either the Mann-Whitney-*U* test for ordinal variables or the Chi-square test for categorical variables, when appropriate.

Uni- and multivariate logistic regression analyses were performed to identify predictors of peri-interventional and long-term stroke in the territory of the treated ICAS. Cox regression analysis was performed to estimate the crude and adjusted (for age and sex) hazard ratio (HR) with 95% confidence intervals (CI). The Kaplan-Meier method and log-rank test were used to detect differences for the cumulative probability of periprocedural and recurrent (ischemic and hemorrhagic) stroke in the territory of the treated artery.

All statistical analyses were performed with SPSS (Version 21.0. IBM Corp., Armonk, NY).

## Results

A total of 126 patients with symptomatic ICAS of the perforator-bearing segments of the MCA M1-segment (59 patients, 1 patient with bilateral M1-stenosis, with a total of 79 treatments) and V4-segment of the VA and/or BA (67 patients with a total of 76 treatments) were treated with PTA alone or Stent-PTA during 01/2007 and 02/2015.

In the posterior circulation group 61 treatments were performed in the BA, 12 in the VA and 3 combined in the VA and BA in one session.

Baseline characteristics are shown in Table 1. Mean age of treated patients was 67 ± 12.1 years. Compared to ICAS in the MCA, patients with a posterior circulation stenosis were significantly more often male (76.3 vs. 49.4%, *p* = 0.001), older (70 ± 8 vs. 61 ± 13.8, *p* < 0.001) and had more often concomitant atrial fibrillation (19.4 vs. 7.8%, *p* = 0.037) treated with oral anticoagulation (22.2 vs. 6.4%, *p* = 0.005). Furthermore, patients with posterior circulation ICAS had significantly more often a stenosis ≥70% (89.5 vs. 72.7%, *p* = 0.008). The symptomatic ICAS was treated within 7 days after the last TIA/stroke in almost one third of all patients with no significant difference between BA/V4 and MCA stenosis (Table 2 in Supplementary Material).

**Table 1:** Baseline patient characteristics and procedural specifications.

	**All treatments**	**MCA**	**BA/V4**	*p*-value^*^
Patients, n	126	59	67	
Total no. of treatments, n	155	79	76	
Treatment for restenosis, n	28	19	9	
Age at time of primary treatment, years, mean (SD; min-max)	67 (12.1; 26–85)	61 (13.8; 26–85)	70 (8.0; 47–83)	<**0.001**
Sex, male, n (%)	97 (62.6)	39 (49.4)	58 (76.3)	**0.001**
Arterial hypertension, n (%)	131 (88.5)	66 (86.8)	65 (90.3)	0.512
Hyperlipidaemia, n (%)	128 (85.9)	64 (83.1)	64 (88.9)	0.312
Diabetes mellitus, n (%)	56 (37.6)	31 (40.3)	25 (34.7)	0.486
Atrial fibrillation, n (%)	20 (13.4)	6 (7.8)	14 (19.4)	**0.037**
Current smoker, n (%)	21 (14.1)	15 (19.5)	6 (8.3)	0.051
Statin before treatment, n (%)	124 (83.8)	64 (84.2)	60 (83.3)	0.885
Oral anticoagulation before treatment, n (%)	21 (14.0)	5 (6.4)	16 (22.2)	**0.005**
Antihypertensive medication before treatment, n (%)	132 (88.6)	66 (85.7)	66 (91.7)	0.253
Degree of stenosis ≥ 70%, n (%)	124 (81.0)	56 (72.7)	68 (89.5)	**0.008**
Procedure ≤ 7 days after last TIA or stroke, n (%)	36 (30)	16 (26.7)	20 (33.3)	0.426
Stent-PTA, n (%)	94 (60.6)	49 (62.0)	45 (59.2)	0.720
Self-expanding stent, n (%)	56 (36.1)	38 (48.1)	18 (23.7)	<**0.001**
Balloon mounted stent, n (%)	38 (24.5)	11 (13.9)	27 (35.5)	**0.001**
Follow-up interval, months, mean (SD, min-max)	19 (23.7; 0–111)	18.5 (24.3; 0–111)	19 (23.5; 0–96)	0.875

* p-values refer to comparison between MCA and BA/V4 treatment. Bold values indicate statistical significance with a p-value < 0.05

Nineteen of the 59 (32.2%) patients with MCA M1-stenosis and 9 of the 67 (13.4%) patients with V4 and/or BA stenosis were treated for restenosis during a mean follow-up of 19 (±23.5) months. PTA alone was performed in 61 and PTAS in 94 of all interventions. There was no significant difference in overall use of stenting between patients with MCA and BA/V4 stenosis (62.0 vs. 59.2%, *p* = 0.72). Self-expanding stents were significantly more often used in the treatment of MCA stenosis (46.8 vs. 25.0%, *p* < 0.001), while balloon-mounted stents were significantly more often used in the straight BA/V4 segments (35.5 vs. 13.9, *p* = 001). Treatment for symptomatic restenosis was performed by PTA alone in 11 cases and PTAS in 15 cases. In two patients treatment of restenosis failed for technical reasons.

### Periprocedural complications and silent DWI lesions

Patients treated for BA/V4 stenosis experienced significantly more often a periprocedural symptomatic infarction compared with patients treated for MCA stenosis [11 (14.5%) vs. 4 (5.1%), *p* = 0.048], while there was no significant difference in periprocedural intracranial hemorrhage [1 (1.3%) vs. 3 (3.8%), *p* = 0.33] or vessel dissection of the treated artery [4 (5.3%) vs. 1 (1.3%), *p* = 0.159]. There were no distal wire perforations observed and the only intraprocedural SAH to occur resulted from a balloon-ruptured vessel with no clinical sequelae. This patient died 3 months after endovascular treatment from a BA thrombosis due to cessation of antiplatelet treatment.

The hazard ratio for a new periprocedural symptomatic infarction adjusted for age and sex in the posterior circulation was 9.53 (95%CI 0.96 to 94.31, *p* = 0.054) compared to the anterior circulation. Periprocedural infarction rate was also significantly higher in patients treated for posterior circulation stenosis when only first interventional treatments were analyzed (14.7 vs. 1.7%, *p* = 0.009). Three out of 4 (75%) periprocedural ischemic strokes in the anterior circulation (one embolic stroke), and 8 out of 11 (72.7%) periprocedural ischemic strokes in the posterior circulation were classified as perforator occlusions, while the remaining 3 periprocedural ischemic strokes in the posterior circulation were classified as mixed perforator and embolic.

**Figure 1:**
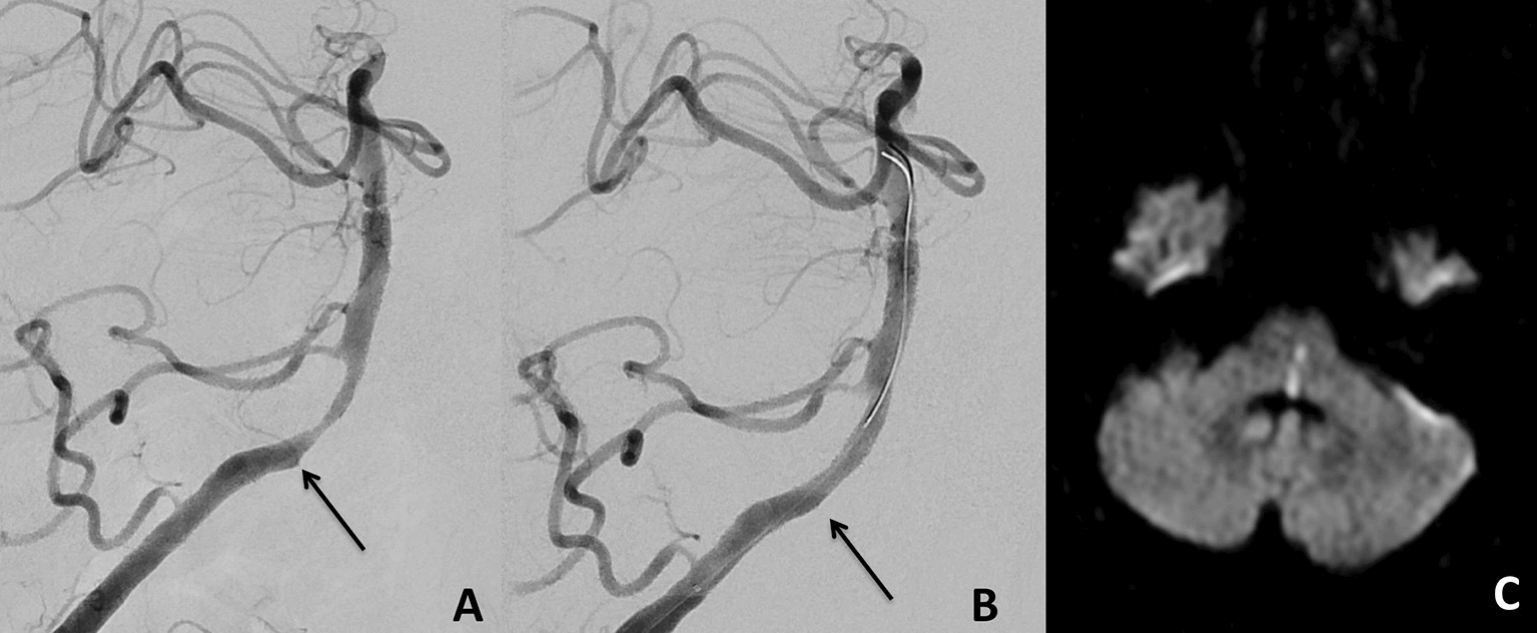
Proximal basilar artery stenosis before treatment **(A)** with the arrow indicating a perforating artery arising at the proximal part of the stenosis. Stent-PTA led to an occlusion of this perforating artery **(B)**. MRI at day 1 after the procedure showed a left paramedian pontomedullary DWI lesion leading to a mRS score of 4 on follow-up examination after 6 months **(C)**.

**Figure 2:**
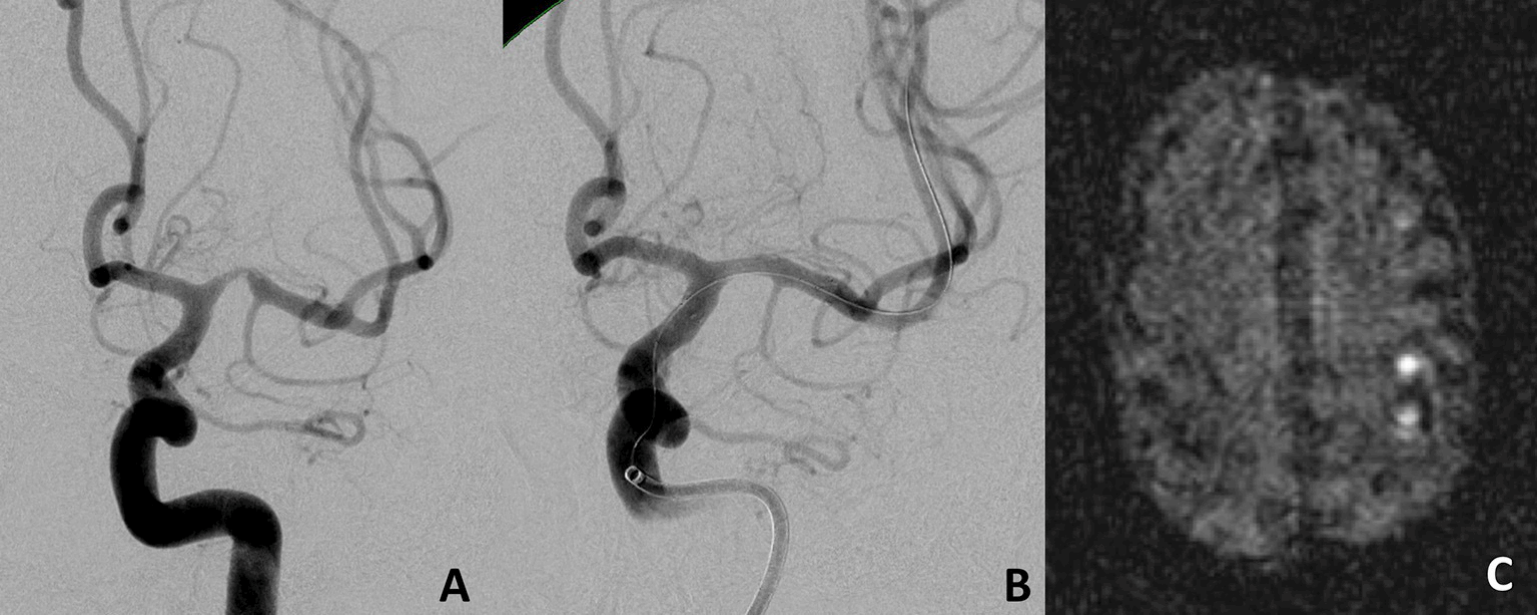
Proximal left middle cerebral artery M1 stenosis before **(A)** and after **(B)** Stent-PTA treatment. MRI at day 1 after the procedure showed asymptomatic cortical embolic infarctions in the left middle cerebral artery territory **(C)**.

Early MRI controls within 3 days post-procedural were available in 76 out of 79 patients in the anterior circulation group and 68 out of 76 patients in the posterior circulation group. New clinically silent DWI lesions were frequently found in both groups with no significant difference between anterior and posterior circulation (23.5% in BA/V4 stenosis vs. 23.6% in M1 stenosis). There were 18 silent DWI lesions in the anterior circulation, thereof 6 in a lenticulostriate perforator territory and 12 cortical embolic in the corresponding MCA territory. In the posterior circulation 3 silent DWI lesions occurred in a medullary or pontine perforator territory and 13 were cortical lesions in a cerebellar or posterior cerebral artery territory. Illustrative cases are shown in Figures 1, 2.

### Long-term follow-up

**Figure 3:**
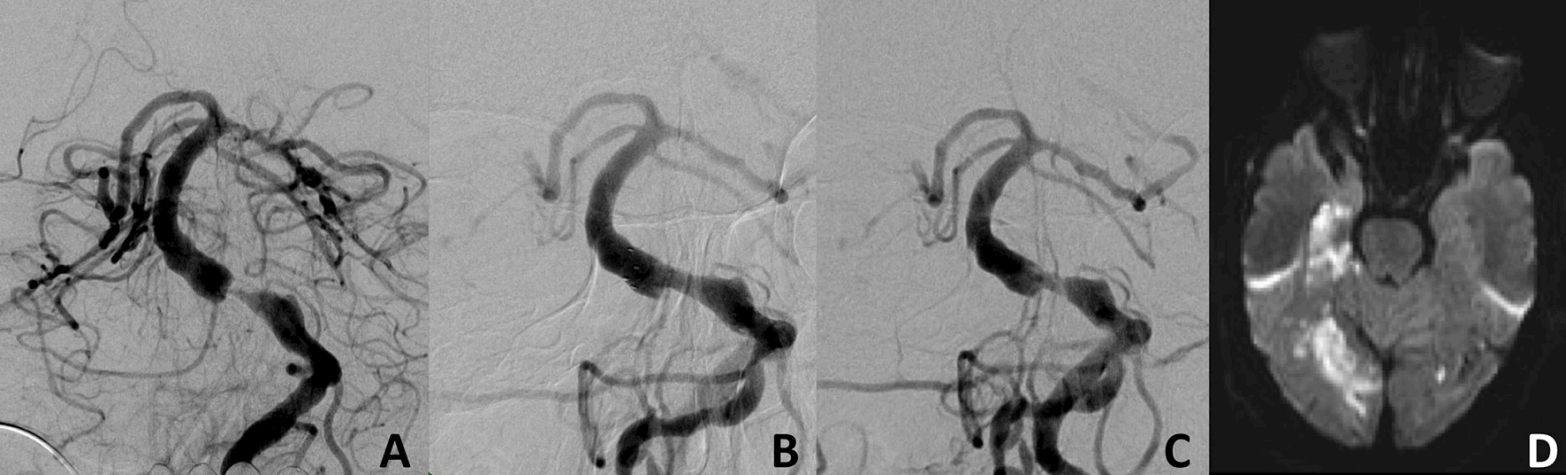
Basilar artery stenosis at the level of the origin of the anterior inferior cerebellar artery before **(A)** and after **(B)** Stent-PTA treatment. In-stent thrombosis after discontinuation of double antiplatelet therapy 4 months after treatment **(C)** with multiple embolic infarcts in the territories of both posterior cerebral arteries **(D)**.

During a mean follow-up period of 19 (±23.7) months, non-significantly more patients treated for BA/V4 stenosis suffered a recurrent ischemic or hemorrhagic stroke in the corresponding vessel territory than patients treated for a MCA M1-stenosis [7 (10.4%) vs. 2 (3.4%), adjusted HR 1.68, 95% CI 0.31–9.10, *p* = 0.549]. Five of the 7 recurrent events in the territory of the BA/V4 stenosis were ischemic strokes, 3 of them classified as delayed stent occlusion and 2 as embolic strokes (see illustrative case in Figure 3). None of the two ischemic strokes occurring during follow-up in the territory of the treated MCA M1-stenosis was classified as perforator occlusion (one delayed stent occlusion and one embolic stroke). Stent occlusion was caused in one patient by cessation of antiplatelet therapy leading to a lethal basilar artery thrombosis. Ischemic strokes in other territories during follow-up occurred in 1 and 2 patients in the anterior and the posterior circulation group, respectively. One patient with a BA stenosis treated by PTA suffered a SAH from a post-stenotic aneurysm 2 months after the procedure and completely recovered after coiling of the aneurysm. Another patient with a BA stenosis treated by PTA experienced an intraparenchymal hemorrhage in the posterior lobe. In three more patients ischemic strokes occurred in other vascular territories due to other aetiologies (i.e., atrial fibrillation). No hemorrhagic stroke occurred during long-term follow-up in patients treated for a MCA M1-stenosis.

### Combined periprocedural and long-term outcome

**Figure 4:**
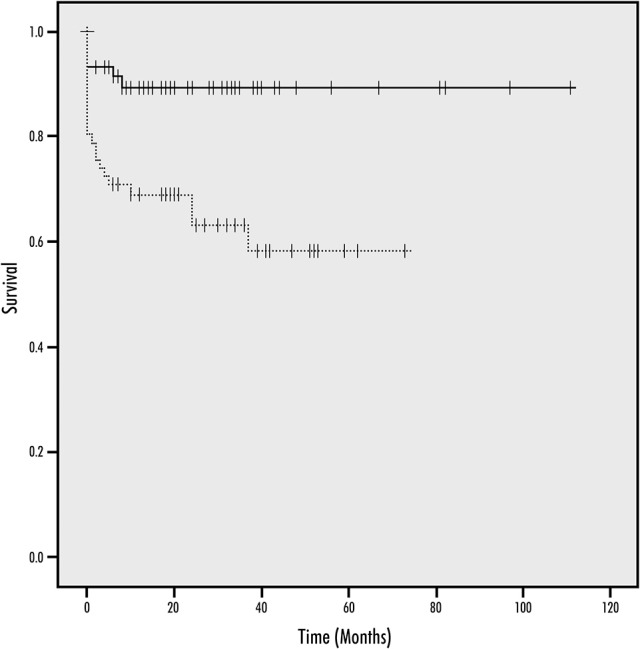
Kaplan-Meier Curves for the cumulative probability of periprocedural ischemic or hemorrhagic stroke and during follow-up (continuous line, anterior circulation; dotted line, posterior circulation; y-axis, survival; x-axis, time in months).

Significantly more patients treated for a BA/V4 stenosis suffered a treatment-related periprocedural or long term ischemic or hemorrhagic stroke than patients treated for a MCA M1-stenosis [19 (25%) vs. 9 (11.4%); adjusted HR 3.78, 95% CI 1.19–12.0, *p* = 0.024]. The Kaplan-Meier curve for the cumulative probability of stroke in the territory of the treated artery is shown in Figure 4.

Multivariate logistic regression analysis showed that a posterior circulation stenosis was the only significant variable of symptomatic brain infarction or hemorrhage in the treated vessel territory during intervention or long-term follow-up (*p* = 0.044). Mortality during follow-up was non-significantly higher in patients with BA/V4 stenosis [6 (11.8%) vs. 1 (1.3%), adjusted HR 3.85, 95% CI 0.41–36.55, *p* = 0.24]. Four of the 7 deaths were clearly related to the PTA/PTAS procedure.

A favorable outcome defined as mRS 0-2 at follow-up was achieved by 88.3% in the anterior and 78.4% in the posterior circulation (adjusted HR 0.74; 95% CI 0.45–1.20, *p* = 0.215).

### Subgroup analysis

There were no significant differences in any periprocedural or long-term outcome parameter between patients treated with PTA alone (*n* = 61) or PTAS (*n* = 94): periprocedural symptomatic infarction 6.6 vs. 11.7% (HR 0.97, 95% CI 0.28–3.34, *p* = 0.957), periprocedural hemorrhage 1.6 vs. 3.2% (*p* = 0.437), dissection 4.9 vs. 2.1% (HR 0.35, 95% CI 0.06–2.13, *p* = 0.256), recurrent stroke in the corresponding vessel territory 9.3 vs. 4.5% (HR 0.47, 95% CI 0.13–1.76, *p* = 0.265), mortality 3.3 vs. 5.3% (HR 1.47, 95% CI 0.29–7.61, *p* = 0.644).

When patients were dichotomized for early (≤7 days, *n* = 36) vs. late (>7 days, *n* = 84; 7 patients with missing information) intervention after the most recent cerebrovascular event, there were also no significant differences in any outcome parameter (periprocedural symptomatic infarction 5.6 vs. 10.8%, HR 2.03, 95% CI 0.42–9.90, *p* = 0.379; periprocedural hemorrhage 2.8 vs. 2.4%, HR 1.28, 95% CI 0.11–14.51, *p* = 0.841; dissection 5.6 vs. 2.4%, *p* = 0.957; recurrent stroke in the corresponding vessel territory 11.8 vs. 4.0%, HR 0.35, 95% CI 0.07–1.63, *p* = 0.179; mortality 8.3 vs. 4.8%, HR 0.69, 95% CI 0.15–3.21, *p* = 0.640).

## Discussion

Given the negative results of the randomized trials on endovascular treatment of symptomatic ICAS [SAMMPRIS (3), VISSIT (4)] and the following decisions of the American Food and Drug Agency (12) and the European Agencies [i.e., the German (13)] to narrow the indications for the use of (the Wingspan) stents for ICAS, we retrospectively examined a large cohort of Caucasian patients with symptomatic ICAS involving intracranial perforator-bearing arterial segments treated with PTA alone or combined PTA and stenting with other stent types. Similar to the randomized trials SAMMPRIS and VISSIT, we observed an overall high rate (22.2%) of ischemic or hemorrhagic symptomatic strokes in the territory of the treated artery, with a significantly higher rate in the posterior circulation. The primary endpoint in SAMMPRIS which included death within 30 days after enrolment occurred in 23% of the patients in the PTAS group during a mean follow-up period of 32.4 months (14). The rate of stroke in the territory of the treated artery in VISSIT was even higher with 34.5% within 1 year of randomization (4). One has to take into account that our stroke rate included 28 recurrent interventional procedures for restenosis of the initially treated ICAS for a total of 155 treatments in our 126 patients, and that our patient cohort was older compared with both SAMMPRIS and VISSIT. The rate of periprocedural infarction was also significantly higher in patients treated for a posterior circulation ICAS when only initial interventional treatments were analyzed in our study. This finding was in line with the detailed analysis of periprocedural strokes in SAMMPRIS (7), a pooled analysis of SAMMPRIS, and VISSIT (5), and an older meta-analysis of case studies (6). In contrast, periprocedural complication rate was not higher in the posterior circulation in the German INTRASTENT multicentric registry (15). However the comparison is limited because recurrence of stroke during long term follow-up was not assessed in INTRASTENT nor in the meta-analysis performed by Gröschel et al. (6, 15).

Three quarters of the periprocedural ischemic strokes in both the anterior and posterior circulation were perforator strokes due to the occlusion of one or more perforating arteries by the atheromatous debris during PTA or stenting (the so called “snow plowing effect”) (16). In contrast, ischemic strokes during long-term follow-up were caused by delayed stent thrombosis of the treated ICAS (partly due to discontinuation of antiplatelet treatment), or embolic strokes in the territory of the treated artery, and not by perforator occlusion. Thus, the mechanism of periprocedural and long-term ischemic stroke is different in patients with ICAS involving perforator segments treated with PTA(S). To our knowledge, there is no published detailed analysis from SAMMPRIS, VISSIT or large case series available which has investigated the etiology of ischemic strokes during long-term follow-up in detail. A possible explanation of the higher rate of periprocedural perforator strokes in the posterior brain circulation is the smaller mean diameter of the perforating arteries (17). Whether high-resolution MRI with visualization of the vessel wall and the atherosclerotic plaque characteristics might result in lower periprocedural ischemic stroke rates in ICAS treatment is questionable since spatial resolution of routinely available 3 Tesla MRI is not able to depict small perforator arteries but only plaque location and contrast enhancement (18, 19). Thus, plaque visualization can be used for image guided treatment planning when it comes to assess an eccentric lesion that is presumably located at the origin of perforating arteries (e.g., the dorsal or dorso-lateral part of the basilar artery).

In contrast to SAMMPRIS, no wire vessel perforation occurred in our study. Distal wire perforations may be associated with difficult exchanges of PTA balloon and stent delivery catheters as shown in the SAMMPRIS analysis with impact of operator and center experience. High enrolling centers had lower rates of hemorrhagic stroke (9.8% at sites enrolling <12 patients vs. 2.7% at sites enrolling ≥12 patients) (8).

A strong point of our study was the rigorous assessment of peri-interventional clinical silent ischemic lesions on DWI MRI after PTA(S), which has only been published in two retrospective Asian case series so far (20, 21). The observed high rate of asymptomatic DWI lesions in one third of our Caucasian patients was in line with the reported 31.7% in 123 Korean patients (20), and lower compared to 46% in 50 Chinese patients (21). Other interventional procedures such as carotid stenting and transcatheter aortic valve implantations have also found such high rates of new embolic brain DWI lesions (22, 23). The clinical impact of these silent ischemic lesions in “non-eloquent” brain areas is still under debate (24), but there is increasing evidence that these embolic MRI lesions might result in subtle neuropsychological sequelae (25). These asymptomatic DWI lesions might have even a higher harmful impact in patients with previous TIA or ischemic stroke and result in future vascular dementia.

The SAMMPRIS investigators hypothesized that PTA alone might substantially lower the risk of periprocedural strokes despite inconsistent study results (14). To date, only a very small randomized trial with 18 included patients compared PTA alone vs. PTAS and did not find a difference in stroke and death rate within 1 month (9). A single-center registry of consecutively treated ICAS patients found a lower stroke rate with PTA alone after 1 year (26), while an older meta-analysis of case series reported a lower 1-year stroke-and/or-death rate with PTAS (27). We did not observe a significant difference in symptomatic stroke rate or asymptomatic DWI lesions between PTA alone or PTAS nor did we detect any complications due to the antiplatelet regimen. However, treatment with PTA and PTAS was not randomized and different stent systems were used in our retrospective study. Balloon-mounted stents were significantly more often used for treatment of posterior ICAS in our study, raising the question, whether this stent type led to the higher complication rate. Only an adequately powered randomized trial against best medical treatment will be able to answer the hypothesis whether PTA alone might substantially lower the risk of periprocedural strokes. Another major limitation of our study is the different long term follow-up periods in our patient cohort.

In conclusion, we have found that PTA alone or PTAS is especially harmful in perforator-bearing segments of the posterior brain circulation due to periprocedural perforator occlusions and delayed stent occlusion or embolic strokes during long term-follow-up. No difference in stroke rate between PTA and PTAS was observed. Silent DWI lesions occurred in almost one third of all treated patients with no difference between the anterior and posterior circulation.

## Author contributions

HN initiated and designed the study, acquired patient data, analyzed and interpreted the data, drafted and revised the paper. He is guarantor. RC initiated the study, analyzed and interpreted data, and revised the draft paper. AA and MW were involved in patient data acquisition, analyzed and interpreted data, and revised the draft paper. MJH, CS, MH, and PH analyzed and interpreted data, and revised the draft paper. RW wrote the statistical analysis plan, acquired, analyzed and interpreted data, and revised the draft paper.

### Conflict of interest statement

The authors declare that the research was conducted in the absence of any commercial or financial relationships that could be construed as a potential conflict of interest.

## Abbreviations

MCA: middle cerebral artery
BA: basilar artery
V4: V4-segment of the vertebral artery
SD: standard deviation
TIA: transitory ischemic attack
PTA: percutaneous transluminal angioplasty.
