# Supplementary material for: Angioplasty and Stenting of Intracranial Arterial Stenosis in Perforator-Bearing Segments: A Comparison Between the Anterior and the Posterior Circulation
Source: Front Neurol. 2018 Jul 9;9:533. doi: 10.3389/fneur.2018.00533 (PMC6046376; doi:10.3389/fneur.2018.00533)
Supplement: Supplementary file 1 [file Data_Sheet_1.docx]

**Table 2:** Details and presumable etiology of ischemic strokes in the territory of the treated vessel occurring periprocedurally and during follow up

| Patient no./  anterior (AC)  or posterior circulation (PC) | Age/Sex | Treated vessel | PTA only or Stent-PTA | Periprocedural stroke (PP) or stroke during follow-up (FU) | Imaging and probable stroke mechanism (perforator occlusion; embolic stroke; delayed vessel or stent occlusion) |
| --- | --- | --- | --- | --- | --- |
| 2 (AC) | 76/m | M1 right | Stent-PTA | Stroke after first treatment during FU | Near complete symptomatic left MCA infarction due to M1 stent occlusion (CT) |
| 20 (AC) | 74/m | M1 right | Stent-PTA | Stroke after first treatment during FU | Symptomatic embolic left MCA infarction (MRI), no in-stent stenosis |
| 26 (AC) | 74/m | M1 left | Stent-PTA | PP stroke during treatment for recurrence | Symptomatic embolic left MCA infarction (MRI) |
| 40 (AC) | 68/f | M1 right | Stent-PTA | PP stroke during treatment for recurrence | Symptomatic infarction in right semioval center and head of caudate nucleus (MRI) |
| 42 (AC) | 61/f | M1 right | Stent-PTA | PP stroke during first treatment | Symptomatic basal ganglia perforator infarction (MRI) |
| 49 (AC) | 43/f | M1 links | Stent-PTA | PP stroke during treatment for recurrence | Symptomatic MCA territory perforator infarction (MRI) |
|  |  |  |  |  |  |
| 1 (PC) | 47/m | BA | PTA only | PP stroke during first treatment | Symptomatic paramedian pontine infarction (MRI) |
| 2 (PC) | 67 m | BA | Stent-PTA | Stroke after first treatment during FU | Complete brainstem infarct due to stent occlusion (CT) |
| 13 (PC) | 74/f | BA | Stent-PTA | PP stroke during first treatment | Symptomatic paramedian pontine infarction (MRI) |
| 14 (PC) | 66/m | BA | Stent-PTA | PP stroke during first treatment | Symptomatic paramedian pontine infarction (MRI) |
| 17 (PC) | 64/m | BA and V4 left | Stent-PTA | PP stroke during first treatment | Symptomatic bilateral cerebellar, right paramedian pontine, left thalamic and left occipital lobe infarction (MRI) |
| 20 (PC) | 70/m | BA | Stent-PTA | Stroke after first treatment during FU | BA occlusion due to stent thrombosis, unsuccessful thrombectomy (DSA, CT) |
| 21 (PC) | 78/m | BA | Stent-PTA | PP stroke during first treatment | Symptomatic bilateral cerebellar and callosal (splenium) infarction(MRI) |
| 22 (PC) | 71/f | BA | Stent-PTA | PP stroke during first treatment | Symptomatic bilateral pontine, mesencephalic and occipital infarction (CT) |
| 25 (PC) | 73/m | BA | Stent-PTA | PP stroke during first treatment | Symptomatic left pontine infarction (MRI) |
| 37 (PC) | 58/m | BA | Stent-PTA | Stroke after first treatment during FU | Symptomatic BA stent thrombosis (DSA, MRI) |
| 41 (PC) | 73/m | BA | PTA only | PP stroke during first treatment | Symptomatic paramedian pontine infarction (MRI) |
| 42 (PC) | 55/m | BA | PTA only | Stroke after first treatment during FU | Symptomatic cerebellar, pontine and occipital lobe infarction (MRI) |
| 45 (PC) | 52/m | BA | PTA only | PP stroke during first treatment | Symptomatic bilateral brainstem and cerebellar infarction (MRI) |
| 56 (PC) | 56/m | V4 right | Stent-PTA | PP stroke during treatment for recurrence | Symptomatic right medulla oblongata infarction (MRI) |
| 62 (PC) | 66/m | BA | PTA only | PP stroke during first treatment | Symptomatic right pontine and left cerebellar infarction (MRI) |
| 66 (PC) | 75/f | V4 left | Stent-PTA | Stroke after first treatment during FU | Symptomatic bilateral cerebellar and occipital lobe infarction (MRI) |

**Abbreviations:** AC = anterior circulation; PC = posterior circulation; BA = basilar artery, M1 = M1-segment of middle cerebral artery, V4 = V4-segment of vertebral artery, FU = follow-up, PP = periprocedural; CT = computed tomography, MRI = magnetic resonance imaging, DSA = digital subtraction angiography
